# CD4 T Helper Cells Instruct Lymphopenia-Induced Memory-Like CD8 T Cells for Control of Acute LCMV Infection

**DOI:** 10.3389/fimmu.2016.00622

**Published:** 2016-12-21

**Authors:** Michaela E. R. Schmitt, Selina Sitte, David Voehringer

**Affiliations:** ^1^Department of Infection Biology, Universitätsklinikum Erlangen, Friedrich-Alexander-University Erlangen-Nürnberg (FAU), Erlangen, Germany

**Keywords:** lymphopenia, memory-like T cells, lymphocytic choriomeningitis virus, PD-1, KLRG1

## Abstract

Lymphopenic conditions lead to expansion of memory-like T cells (T_ML_), which develop from naïve T cells by spontaneous proliferation. T_ML_ cells are often increased in the elderly population, AIDS patients, and patients recovering from radio- or chemotherapy. At present, it is unclear whether T_ML_ cells can efficiently respond to foreign antigen and participate in antiviral immunity. To address this question, we analyzed the immune response during acute low-dose infection with lymphocytic choriomeningitis virus-WE in T cell lymphopenic CD4Cre/R-diphtheria toxin alpha (DTA) mice in which most peripheral T cells show a T_ML_ phenotype. On day 8 after infection, the total number of effector T cells and polyfunctional IFN-γ and TNF-α producing CD8 T cells were three- to fivefold reduced in CD4Cre/R-DTA mice as compared to controls. Viral clearance and the humoral immune response were severely impaired in CD4Cre/R-DTA mice although CTLs efficiently killed transferred target cells *in vivo*. Transfer of naïve CD4 T cells but not anti-PD-L1 blockade restored the expansion of antigen-specific polyfunctional CD8 T cells and resulted in lower viral titers. This finding indicates that under lymphopenic conditions endogenous CD4 T_ML_ cell lack the capacity to promote expansion of CTLs. However, CD8 T_ML_ cells retain sufficient functional plasticity to participate in antiviral immunity in the presence of appropriate help by fully functional CD4 T cells. This capacity might be exploited to develop treatments for improvement of CD8 T cell functions under various clinical settings of lymphopenia.

## Introduction

T cell homeostasis is regulated by thymic output as well as proliferation and death of peripheral T cells (1). Naïve T cells require recognition of cognate MHC and cytokines like IL-7 or IL-15 for survival. Memory T cells (T_M_) are less dependent on cognate MHC but IL-7 and IL-15 mediated signals increase their lifespan (2). The total number of peripheral T cells and the ratio of naïve to T_M_ remain remarkably stable until the seventh decade of life (3). Above that age T cell numbers decrease and certain T cell clones expand resulting in a lymphopenic immune system with an oligoclonal repertoire of TCR specificities and a relative increase in the pool of “memory phenotype” T cells (T_MP_). The pool of T_MP_ cells consists of real antigen-experienced T_M_ and memory-like T cells (T_ML_) that converted from naïve T cells by spontaneous proliferation under lymphopenic conditions [lymphopenia-induced proliferation (LIP)] (4, 5). Increased numbers of T_ML_ cells are believed to make a substantial contribution to the T_MP_ pool in AIDS patients or patients recovering from radio- or chemotherapy. However, it remains unclear whether T_ML_ cells can be engaged in mounting a primary immune response. T_ML_ cells may further compete with real T_M_ for survival, which could lead to attrition of the T_M_ pool under lymphopenic conditions (6–8).

Lymphopenia-induced proliferation requires recognition of self-peptide/MHC complexes but occurs independently of costimulatory signals (9, 10). For CD4 T cells, it could be shown that LIP can be subdivided in a fast, IL-7-independent and probably DC-dependent proliferative response (“spontaneous” or “endogenous” proliferation) and a slower, IL-7-dependent and probably DC-independent response (“homeostatic” proliferation) (11, 12). Lymphopenia is a risk factor for autoimmunity since self-reactive T cells can expand and initiate autoimmune reactions under lymphopenic conditions (13–15). Autoimmunity can develop in HIV patients under highly active anti-retroviral therapy due to expansion of autoreactive T cells (16). The elderly population often shows poor responses to vaccines and has a high frequency of T_MP_. Therefore, it is important to study the functionality of T_ML_ cells to develop more efficient vaccination strategies for the elderly population and to characterize peripheral tolerance mechanisms that control the onset of autoimmunity (17).

Although it remains difficult to estimate the contribution of T_ML_ cells to the total pool of memory T cells (T_MP_), it has recently been shown that the T_MP_ population in germ-free mice contains T_ML_ cells, which demonstrates that T_ML_ cells are not dependent on the gut flora (18). For CD8 T cells, it has been shown that T_ML_ and antigen-experienced T_M_ are basically indistinguishable by phenotype or gene expression profile (1, 19) although more recent data showed that CD8^+^ T_ML_ cells differ from true-memory cells by expression of different chemokine receptors (6). Some studies indicated that CD8^+^ T_ML_ cells are functional and provide protective immunity (6, 20). Furthermore, it was shown that protection against Listeria-OVA by OT-I T_ML_ cells that were generated by transfer into irradiated recipient mice required a low number of CD4 T cells (even antigen non-specific cells worked), irradiation-induced bacteremia, and IL-12 (21). Far less is known about T_ML_ cells within the CD4^+^ T cell compartment.

We previously generated mice in which most peripheral T cells show a memory phenotype (22). In this model, naïve T cells are deleted due to diphtheria toxin alpha (DTA) expression from the Rosa26 locus after Cre-mediated excision of a loxP-flanked stop cassette. These CD4Cre/R-DTA mice have severely reduced naïve (CD62L^+^CD44^−^) CD4 and CD8 T cells in peripheral lymphoid tissues due to DTA expression in developing T cells in the thymus (22). However, some T cells escape this toxin-mediated deletion, undergo homeostatic proliferation and develop into T_ML_ cells which fill the T_MP_ niche to the same level as in control mice while naïve CD4 and CD8 T cells remain constitutively deleted.

Here, we used this mouse model to determine the functionality of T_ML_ cells in response to acute low-dose infection with lymphocytic choriomeningitis virus (LCMV)-WE. LCMV-infected CD4Cre/R-DTA mice efficiently killed transferred target cells *in vivo* in an antigen-specific manner. However, the infection-induced expansion of LCMV-specific T cells, viral clearance, and the humoral immune response were severely impaired in CD4Cre/R-DTA mice as compared to control mice. Transfer of polyclonal naïve CD4 T cells from wild-type mice but not anti-PD-L1 blockade restored the expansion and function of endogenous CD8 T_ML_ cells in CD4Cre/R-DTA mice.

## Materials and Methods

### Mice and Infection

Homozygous CD4Cre mice (23) were crossed to R-DTA mice (22) to generate lymphopenic CD4Cre^+^R-DTA^+^ mice (CD4Cre/R-DTA) and CD4Cre^+^R-DTA^−^ control mice (CD4Cre). B6_CD45.1 congenic mice (B6.SJL-Ptprca Pepcb/BoyJ) were originally obtained from The Jackson Laboratory and crossed to normal C57BL/6J mice to generate heterozygous CD45.1^+^CD45.2^+^ congenic mice. C57BL/6J mice were purchased from Charles River Laboratories (Sulzfeld, Germany). Mice were maintained in the Franz-Penzoldt-Zentrum in Erlangen under specific pathogen-free conditions. Mice were infected with 200 pfu of LCMV-WE intravenously under biosafety level 2 and analyzed at indicated points in time. All experiments were performed in accordance with German animal protection law and European Union guidelines 86/809 and were approved by the Federal Government of Lower Franconia.

### Flow Cytometry

Single-cell suspensions of spleens were generated under biosafety level 2 by mechanical disruption and erythrocytes were lysed with ACK-buffer (0.15 M NH_4_Cl, 1 mM KHO_3_, 0.1 mM Na_2_EDTA). Cells were preincubated with anti-CD16/CD32 mAb (clone 2.4G2; BioXcell, West Lebanon, NH, USA) and stained with respective antibodies. The following antibodies were used for surface staining: PerCP-Cy5.5- or APCe780-labeled anti-CD4 (clone RM4-5), FITC-, PE-, or APC-labeled anti-CD8 (clone 53-6.7), PE-Cy7-labeled anti-CD62L (clone MEL-14), eFluor660-labeled anti-GL-7 (clone GL-7), FITC- or eFluor450-labeled anti-CD45R (clone RA3-6B2), FITC-labeled anti-CD44 (clone IM7), e450-labeled anti-KLRG1 (clone 2F1), PerCP-labeled anti-CD45.2 (clone 104), and PE- or e450-labeled anti-CD45.1 (clone A20) were purchased from eBioscience (San Diego, CA, USA). PE-Cy7-labeled anti-CD38 (clone 90), PE-Cy7-labeled anti-CD4 (clone RM4-5), and PE-Cy7-labeled or biotinylated anti-PD-1 (clone RMP1-30) were purchased from BioLegend (San Diego, CA, USA). Vioblue- or APC-labeled anti-CD44 (clone IM7.8.1) was purchased from Miltenyi Biotec (Bergisch Gladbach, Germany), PE-labeled anti-CXCR5 (clone 2G8) and V500-labeled Streptavidin were from BD Biosciences (San Jose, CA, USA).

For dextramer stainings (gp33_H2-D^b^ coupled to APC; Immudex, Copenhagen, Denmark), cells were washed with PBS containing 5% FCS, incubated with 5 µl dextramer per sample for 10 min at room temperature and then the antibody mixture for surface staining was added for an additional 20 min at 4°C. Tetramer staining (gp66_I-A^b^ coupled to PE, NIH tetramer core facility) was performed in RPMI1640 (PAN-Biotech, Aidenbach, Germany) containing 10% FCS. Cells were incubated with 0.3 ng tetramer for 2 h at 37°C, washed, and stained with respective antibodies.

FITC-labeled anti-mouse IFN-γ (clone XMG1.2; BioLegend) and PE-labeled anti-mouse TNF-α (clone MP6-XT22; eBioscience) were used for intracellular staining after cells had been fixed with 4% paraformaldehyde and permeabilized with the Intracellular Staining Perm Wash Buffer (BioLegend) according to the manufacturer’s protocol. Dead cells were excluded by staining with DAPI (Sigma-Aldrich, St. Louis, MO, USA), fixable viability dye APC-eFluor780, or fixable viability dye APC-eFluor506 (both from eBioscience). Samples were acquired with FACS Canto II (BD Bioscience) and MACSQuant (Miltenyi Biotec). Data were analyzed using FlowJo software (TreeStar, Ashland, OR, USA).

### Restimulation of T Cells

Single-cell suspensions were either restimulated with 1 µg/ml gp33- (KAVYNFATM) or gp61- (GLKGPDIYKGVYQFKSVEFD) peptide (JPT, Berlin, Germany) for 4 h. After 2 h, 10 µg/ml Brefeldin A (Sigma-Aldrich, St. Louis, MO, USA) was added. IFN-γ and TNF-α production was measured by intracellular staining.

### Quantitative RT-PCR

RNA was prepared from the indicated organs with the RNeasy Mini Kit (Qiagen, Hilden, Germany) according to the manufacturer’s protocol. To transcribe the viral RNA genome, 1 µg of total RNA was reversely transcribed into cDNA with the LCMV-gp-specific reverse primer. Quantitative PCR was performed with SYBR Select Master Mix (Thermo Fisher Scientific, Waltham, MA, USA), and the following primer sequences: LCMV-gp forward primer 5′-CATTCACCTGGACTTTGACAGACTC-3′ and LCMV-gp reverse primer 5′-GCAACTGCTGTGTTCCCGAAAC-3′ under the following conditions: 95°C 30 s, 60°C 20 s, 72°C 20 s; 40 cycles. Copy numbers of LCMV genomes per gram organ were determined using a plasmid containing 115 bp of LCMV gp gene.

### *In Vivo* Cytotoxicity Assay

Splenocytes from naïve B6 mice were stained with 5 µM CellTraceViolet (Thermo Fisher Scientific), loaded with 1 µM gp33 peptide (JPT), and mixed with splenocytes from CD45.1 congenic mice loaded with 1 µM m45 peptide of MCMV (JPT). The 4.5 × 10^6^ cells per group were mixed and transferred into LCMV-infected or naïve mice at day 8 after infection. Analysis took place 90 min after cell transfer.

### Adoptive T Cell Transfers

CD4 or CD8 T cells from spleens of CD45.1 congenic mice were isolated using the EasySep isolation Kit (STEMCELL Technologies, Vancouver, BC, Canada) according to manufacturer’s protocol. Then, 3 × 10^6^ CD4 or CD8 T cells were transferred to recipient mice 1 day before LCMV infection. Mice were analyzed on indicated days after infection.

### ELISA

Plates were coated with 1 µg/ml nucleoprotein of LCMV (Alpha Diagnostics, San Antonio, TX, USA), blocked with PBS + 1% BSA and incubated with sera overnight. Alkaline phosphatase-coupled anti-mouse IgM, anti-mouse IgG1, or anti-mouse IgG2c antibodies and para-nitrophenylphosphate substrate (both Southern Biotec, Birmingham, AL, USA) were used for detection. ELISA was measured at 405 nm with Multiskan FC multiplate photometer (Thermo Fisher Scientific).

### Immunofluorescence Staining

To analyze the germinal centers, cryosections of spleens were dehydrated in −20°C cold acetone and rehydrated in PBS. Germinal centers were stained with biotinylated anti-mouse CD4 (clone GK1.5, eBioscience), APC-labeled anti-mouse CD45R (clone RA3-6B2, eBioscience), and FITC-labeled GL-7 (clone GL-7, eBioscience) followed. As secondary antibody we used Streptavidin-Cy3 (Jackson Immunoresearch, West Grove, PA, USA). Nuclei were counterstained with DAPI.

### Statistics

Student’s *t*-test was used for normally distributed (Shapiro–Wilk test) data sets whereas Mann–Whitney *U*-test was used for non-normally distributed data sets. Analysis was performed with SigmaPlot (Systat Software Inc., San Jose, CA, USA) software, and *P*-values of less than 0.05 were considered statistically significant.

## Results

### Impaired T Cell and Humoral Response to LCMV in CD4Cre/R-DTA Mice

**Figure 1:**
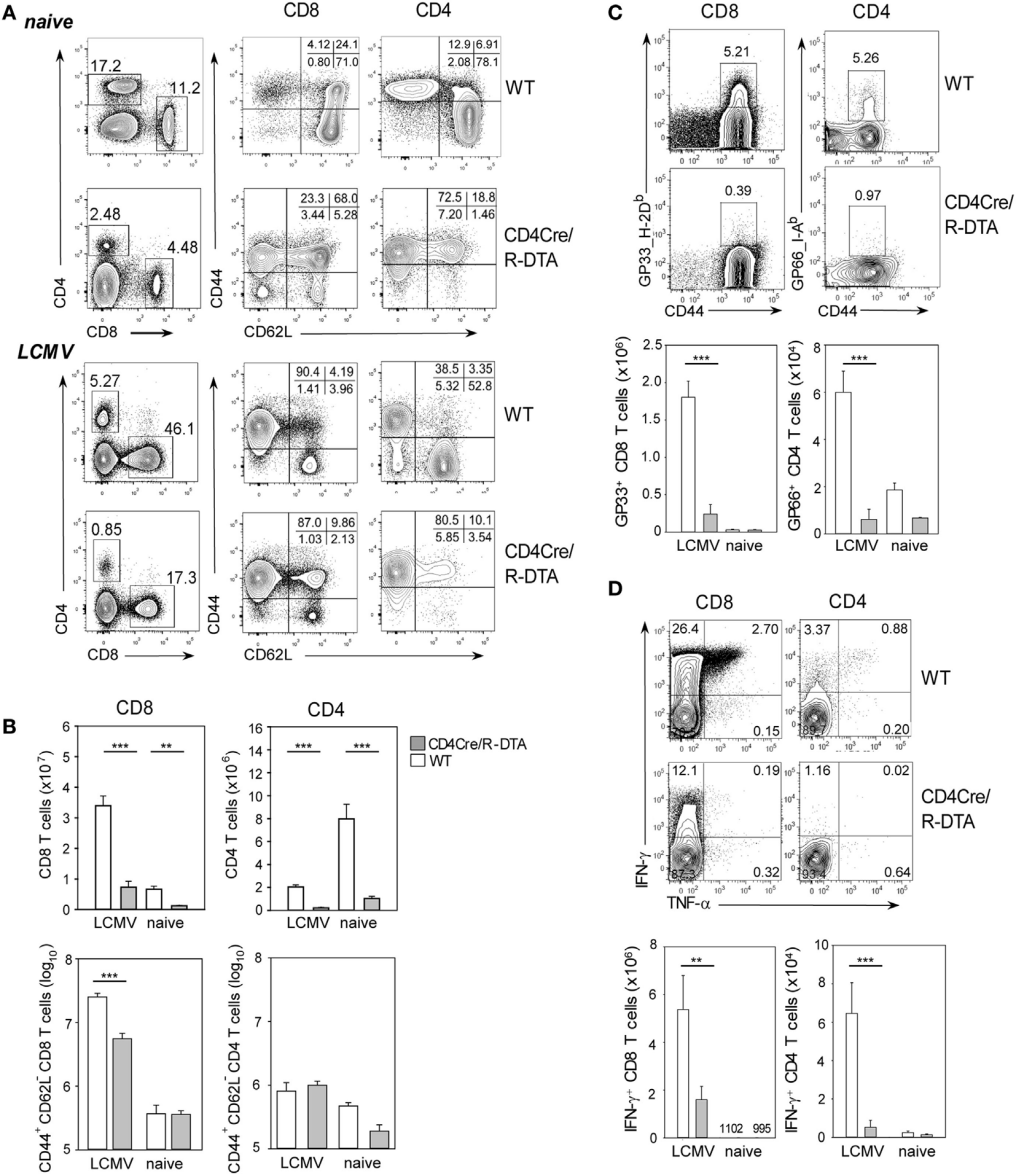
**Impaired T cell response to LCMV in CD4Cre/R-diphtheria toxin alpha (DTA) mice**. Spleens of CD4Cre/R-DTA mice and CD4Cre control mice were analyzed by flow cytometry before (naïve) or 8 days after (infected) lymphocytic choriomeningitis virus-WE infection. **(A)** Contour plots show representative stainings of the total CD8 and CD4 T cells and their activation status. **(B)** Bar graphs depict the mean number + SEM of activated (CD44^+^CD62L^−^) and total CD8 or CD4 T cells in CD4Cre (white) and CD4Cre/R-DTA mice (gray). **(C)** Contour plots show stainings with gp33 dextramer (left panel) on gated CD8 T cells or gp66 tetramer (right panel) on gated CD4 T cells. Bar graphs depict the mean number + SEM of gp33-specific CD8 T cells or gp66-specific CD4 T cells in LCMV-infected or naïve CD4Cre (white) and CD4Cre/R-DTA mice (gray). **(D)** Intracellular staining of IFN-γ and TNF-α in gated CD8 T cells restimulated with gp33 peptide (left panel) or in gated CD4 T cells restimulated with gp61 peptide (right panel). Bar graphs depict the mean number + SEM of IFN-γ producing CD8 or CD4 T cells in LCMV-infected or naïve CD4Cre (white) and CD4Cre/R-DTA mice (gray). A total of 2–15 naïve and at least 8 LCMV-infected mice per group from several independent experiments. **P < 0.01, ***P < 0.001 by Mann–Whitney *U*-test.

Expression of DTA in developing T cells of CD4Cre/R-DTA mice leads to almost complete deletion of naïve peripheral T cells despite normal numbers of T cells with a memory phenotype (T_MP_) [(22); Figure 1A]. This indicates that the niche for T_MP_ cells had been completely filled by homeostatically proliferated T_ML_ cells that escaped deletion and retained the loxP-flanked stop cassette in front of DTA in the Rosa26 locus. To determine whether a T_ML_-dominated immune system is able to mount a protective antiviral immune response, we infected CD4Cre/R-DTA mice and CD4Cre control mice with 200 pfu LCMV-WE and analyzed the T cell populations in the spleen on day 8 after infection. Both CD4Cre/R-DTA and control mice showed massive (~20- to 30-fold) expansion of effector CD8 T cells (CD44^+^CD62L^−^) in the spleen although the total number of effector CD8 T cells was fivefold lower in CD4Cre/R-DTA mice as compared to control mice (Figure 1; Figure S1 in Supplementary Material). By staining for antigen-specific T cells using gp33_H-2D^b^ dextramers and gp66_I-A^b^ tetramers that cover immunodominant CD8 and CD4 T cell epitopes, respectively, we observed about 10-fold fewer CD8 and CD4 T cells specific for these epitopes in CD4Cre/R-DTA mice as compared to controls (Figure 1C). *In vitro* restimulation with LCMV peptides further revealed a significantly impaired production of IFN-γ and/or TNF-α in T cells of LCMV-infected CD4Cre/R-DTA mice (Figure 1D). The larger number of cytokine-producing as compared to gp33_H-2D^b^ dextramer positive cells could be due to a contribution of CD8 T cells that recognized the gp33 peptide in H-2K^b^. An alternative explanation would be that the dextramer staining did not detect all gp33_H-2D^b^-specific T cells due to downregulation of the TCR.

**Figure 2:**
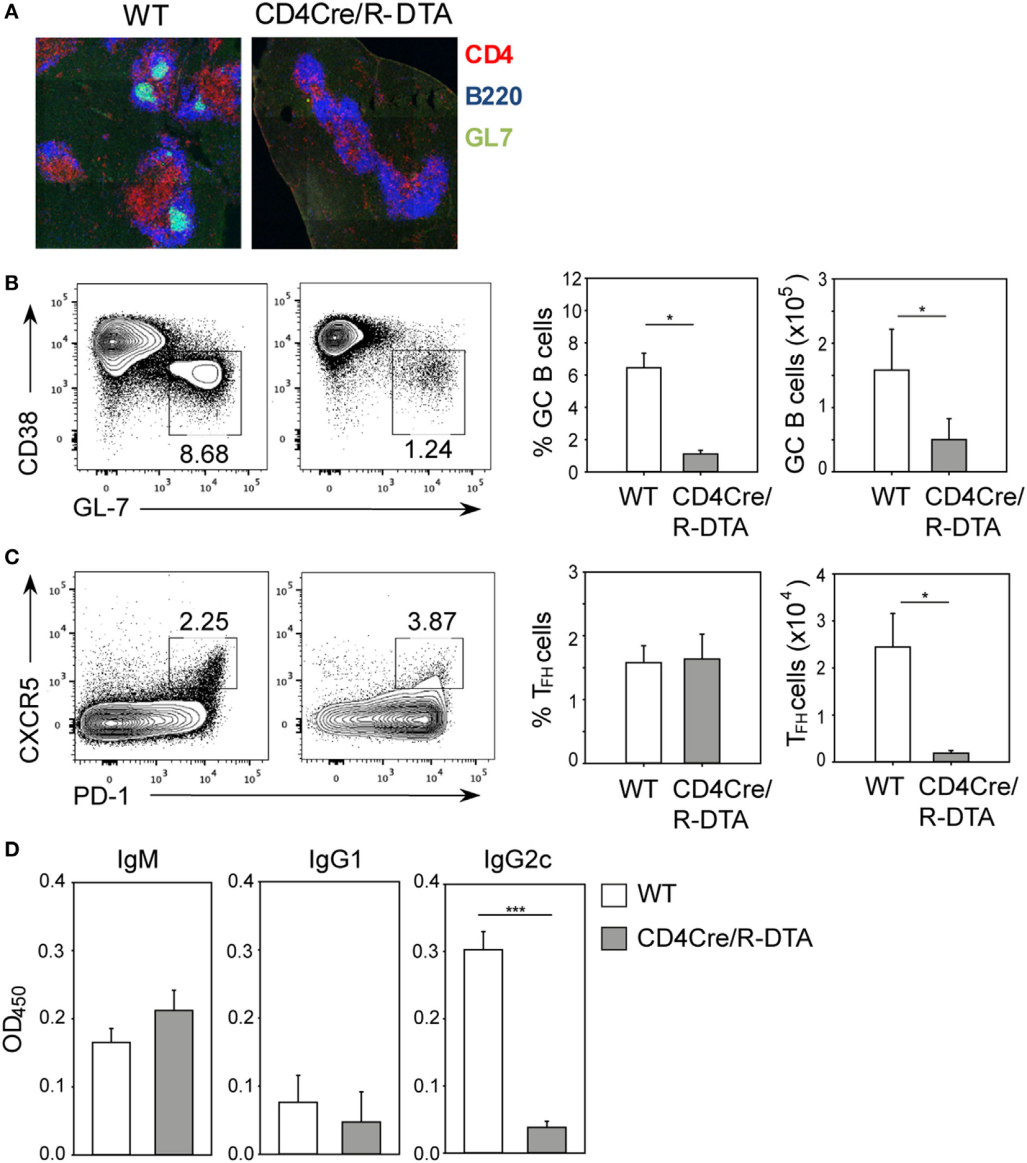
**Impaired humoral immune response to lymphocytic choriomeningitis virus (LCMV) in CD4Cre/R-diphtheria toxin alpha (DTA) mice**. CD4Cre and CD4Cre/R-DTA mice were infected with lymphocytic choriomeningitis virus-WE and spleens and sera were analyzed on day 14 after infection. **(A)** Spleen sections were stained with anti-CD4 (red), anti-B220 (blue), and GL-7 (green) to visualize the germinal centers. **(B)** Contour plots show the percentage of GC B cells (CD38^lo^GL-7^hi^ gated on B220^+^) in the spleen of indicated LCMV-infected mice. Bar graphs depict the mean percentage + SEM (left) or mean number + SEM (right) of GC B cells in LCMV-infected CD4Cre (white) and CD4Cre/R-DTA mice (gray). **(C)** Contour plots show the percentage of T follicular helper (T_FH_) cells (PD-1^hi^CXCR5^hi^ gated on CD4^+^) in the spleen of indicated LCMV-infected mice. Bar graphs depict the mean percentage + SEM (left) or mean number + SEM of T_FH_ cells in LCMV-infected CD4Cre (white) and CD4Cre/R-DTA mice (gray). A total of 4–17 mice per group from 1 to 4 experiments. **(D)** LCMV nucleoprotein-specific IgM, IgG1, and IgG2c antibody levels in sera from LCMV-infected CD4Cre (white) and CD4Cre/R-DTA mice (gray). Bar graphs show the mean + SEM of 9–12 mice per group from 3 experiments. *P < 0.05, ***P < 0.001 by Mann–Whitney *U*-test.

Although CD8 T cells are critical for control of acute LCMV infection, antibodies have been shown to contribute to long-term protection of LCMV infection by preventing viral spread and by maintaining functional CTL memory (24–26). We reasoned that in CD4Cre/R-DTA mice, the humoral immune response might be affected by the reduced number of CD4 T cells which are critical for providing help to B cells. Indeed, the formation of germinal centers in LCMV-infected CD4Cre/R-DTA mice was abolished (Figure 2A). This correlated with impaired expansion of T follicular helper (T_FH_) cells identified as CD4^+^PD-1^+^CXCR5^+^ cells and germinal center B cells identified as B220^+^CD38^−^GL-7^+^ cells (Figures 2B,C). Furthermore, the serum levels of LCMV-specific IgG2c antibodies were reduced in CD4Cre/R-DTA mice while IgM and IgG1 titers appeared comparable between both groups of mice (Figure 2D).

### Lack of Viral Control Despite Efficient *In Vivo* Killing of Transferred Target Cells

**Figure 3:**
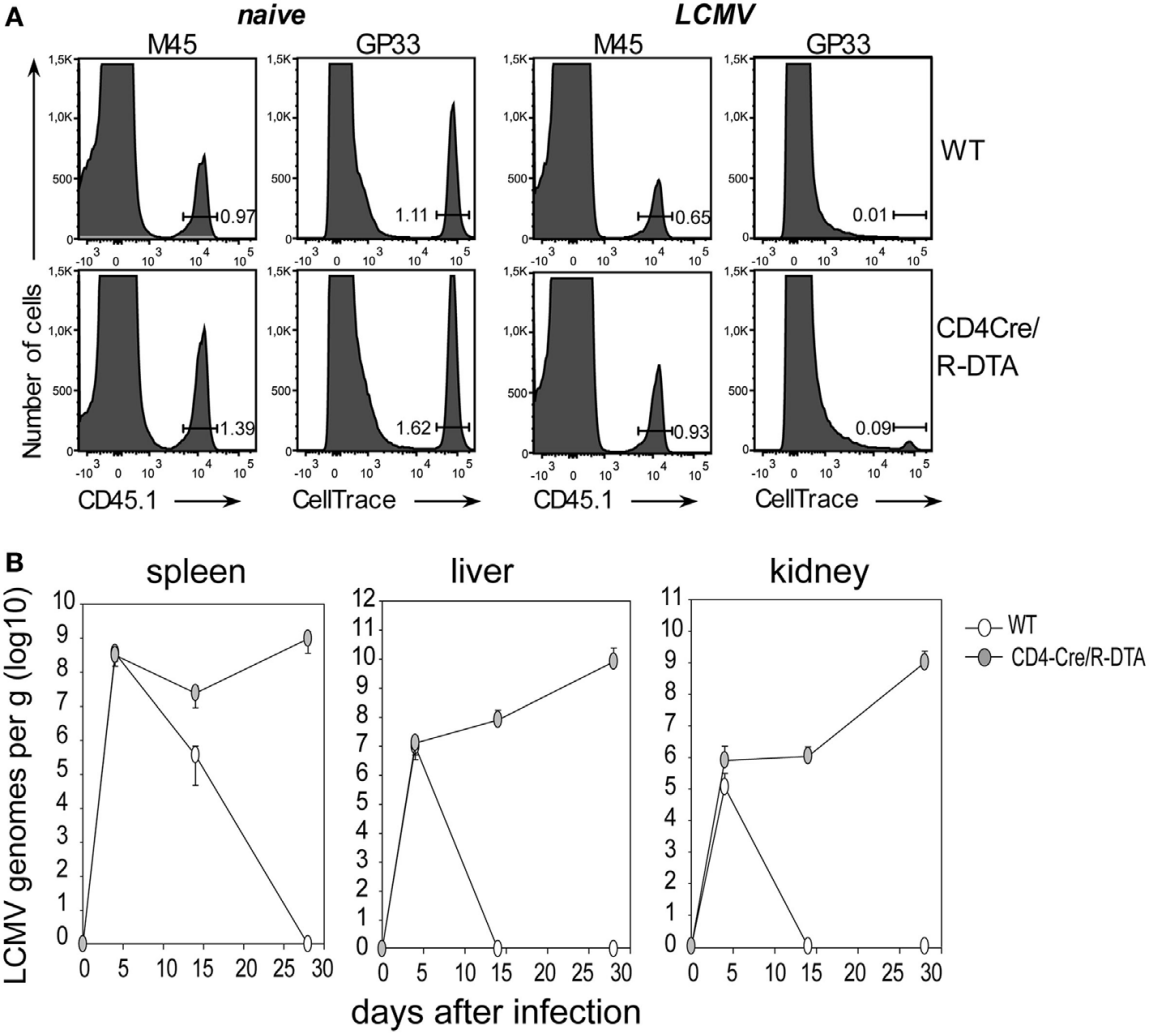
**Lack of viral control despite efficient *in vivo* killing of transferred target cells**. **(A)** *In vivo* killing assay. Lymphocytic choriomeningitis virus (LCMV)-infected or naïve CD4Cre and CD4Cre/R-diphtheria toxin alpha (DTA) mice received LCMV-gp33 peptide-loaded, CellTrace-labeled splenocytes together with control cells (CD45.1^+^) loaded with an irrelevant peptide (m45 from murine cytomegalovirus). Histograms show the frequency of indicated target cells at 90 min after cotransfer into day 8 LCMV-infected mice. Histograms are representative for three mice per group from two experiments. **(B)** CD4Cre (white) and CD4Cre/R-DTA mice (gray) were infected with LCMV and the copy numbers of viral genomes in spleen, liver, and kidney were determined by quantitative RT-PCR at the indicated days after infection. Data points show the mean copy number + SEM of three to five mice per group per timepoint.

With the next set of experiments we compared the *in vivo* killing capacity of CTLs, and the control of viral load in LCMV-infected CD4Cre/R-DTA and control mice. The *in vivo* killing capacity was determined by cotransfer of two differently marked target cells into day 8 LCMV-infected recipient mice. One set of target cells consisted of syngeneic splenocytes labeled with fluorescent CellTrace dye and loaded with LCMV-gp33 peptide while the other set of target cells consisted of MCMV-M45 control peptide-loaded splenocytes from congenic CD45.1 mice. The frequency of transferred target cells in the spleen was analyzed 90 min later by flow cytometry. We observed that the specific killing efficiency was comparable in both strains of mice (Figure 3A). Based on this result, we expected that CD4Cre/R-DTA mice would be able to control the infection. Therefore, we analyzed the copy numbers of LCMV genomes in spleen, liver, and kidney on day 4, 14, and 28 after infection by quantitative RT-PCR. Control mice eliminated the virus from liver and kidney within 14 days and from the spleen within 28 days. By contrast, CD4Cre/R-DTA mice were not able to control the infection and copy numbers of LCMV genomes even increased at 28 days after infection (Figure 3B).

### Anti-PD-L1 Block Does Not Rescue the Functionality of CD8 T_ML_ Cells

**Figure 4:**
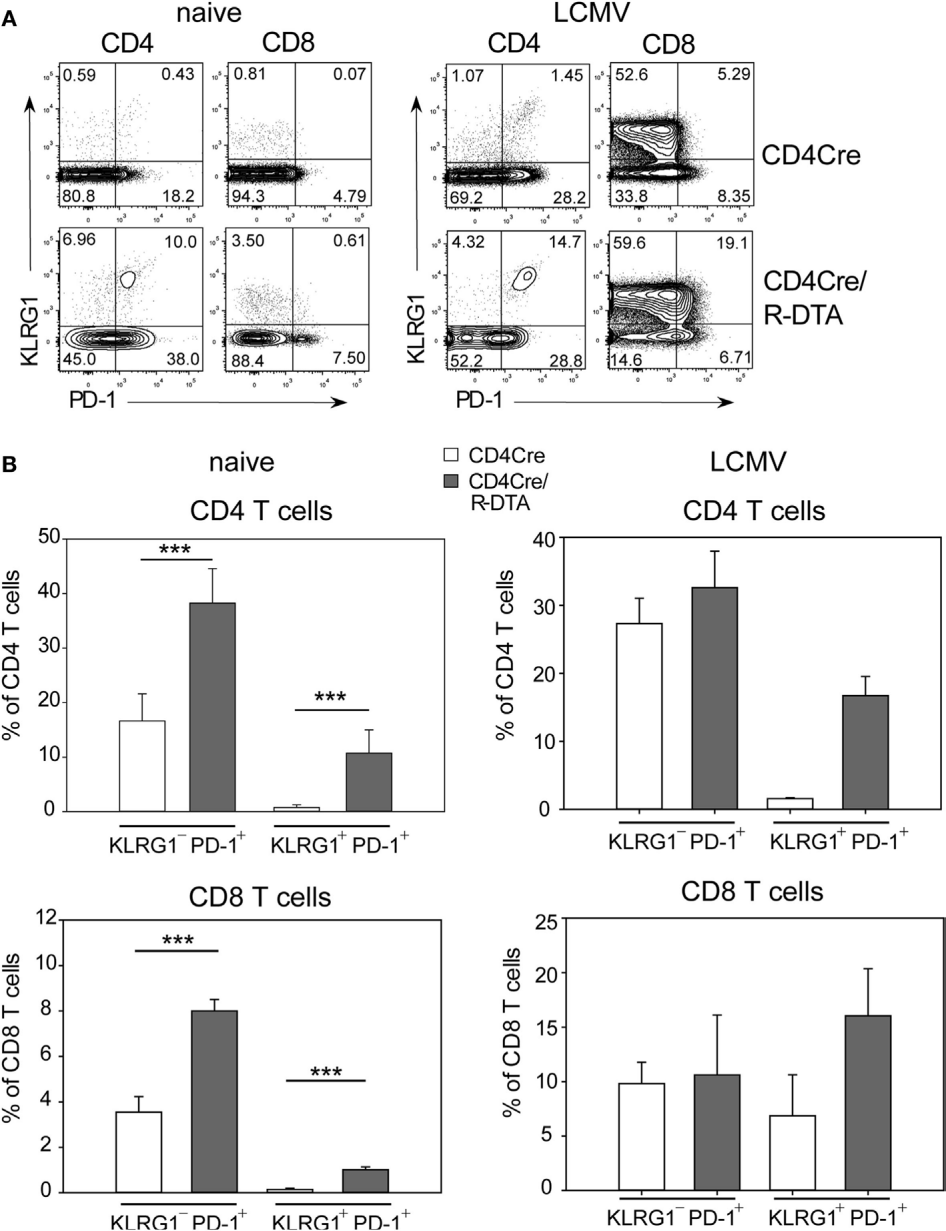
**Expression of KLRG1 and PD-1 on T cells of naïve CD4Cre/R-diphtheria toxin alpha (DTA) mice**. **(A)** Dot plots show the expression of KLRG1 and PD-1 on gated CD4 or CD8 T cells from naïve or lymphocytic choriomeningitis virus (LCMV)-infected CD4Cre/R-DTA and CD4Cre mice. **(B)** Bars show the mean + SEM frequency of KLRG1^+^PD-1^+^ and KLRG1^−^PD-1^+^ T cells among gated CD4 or CD8 T cells from naïve or LCMV-infected CD4Cre/R-DTA and CD4Cre mice. Naïve: 9–16 mice per group from three experiments. LCMV-infected: two to four mice per group from one experiment. ***P < 0.001 by Mann–Whitney *U*-test.

**Figure 5:**
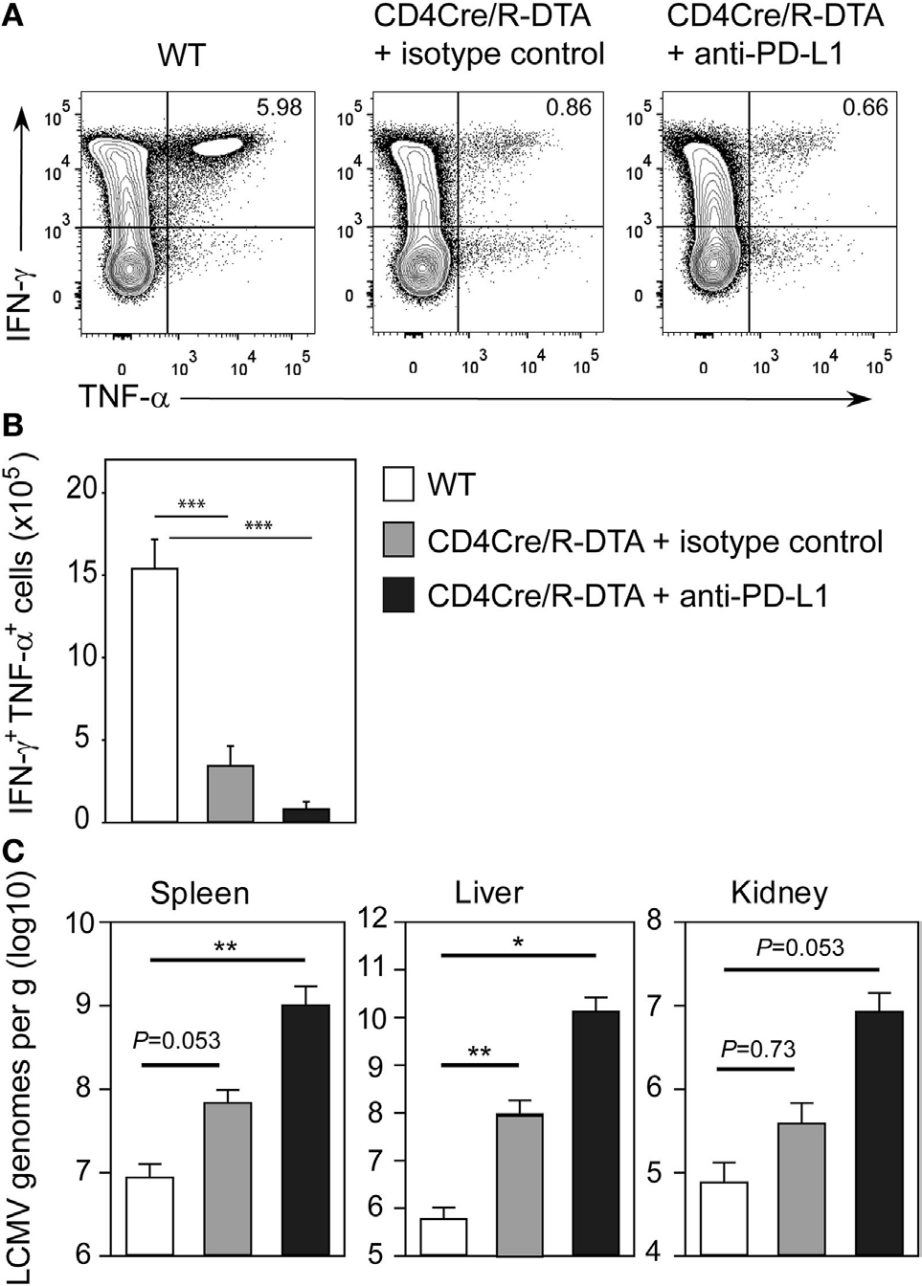
**Anti-PD-L1 block does not restore the functionality of CD8 memory-like T cells**. CD4Cre and CD4Cre/R-diphtheria toxin alpha (DTA) mice were infected with lymphocytic choriomeningitis virus (LCMV). CD4Cre/R-DTA mice were injected with 200 µg anti-PD-L1 antibody or isotype control i.p. at day 1, day 4, and day 7 after infection. Spleens were analyzed at day 10 after infection. **(A)** Contour plots show intracellular stainings of IFN-γ and TNF-α in gated CD8 T cells restimulated with gp33 peptide. **(B,C)** Bar graphs depict the mean number + SEM of IFN-γ and TNF-α producing CD8 T cells **(B)** or copy numbers of viral genomes in spleen, liver, and kidney determined by quantitative RT-PCR **(C)** in LCMV-infected CD4Cre mice (white), LCMV-infected isotype control-treated CD4Cre/R-DTA mice (gray), and LCMV-infected anti-PD-L1-treated CD4Cre/R-DTA mice (black). Six to seven mice per group from two experiments. *P < 0.05, **P < 0.01, ***P < 0.001 by Mann–Whitney *U*-test.

The lack of viral control and impaired expansion of LCMV-specific effector T cells in CD4Cre/R-DTA mice raised the question whether expression of inhibitory receptors on T_ML_ cells might dampen their responsiveness as previously suggested by others (27). Indeed, a larger fraction of T cells expressed the inhibitory receptor PD-1 alone or in combination with the senescence marker KLRG1 in naïve CD4Cre/R-DTA mice as compared to control mice (Figure 4A). Expression of both markers increased after LCMV infection in both strains of mice (Figure 4B). The expression of PD-1 on CD8 T cells has also been observed in chronically LCMV-infected mice and correlated with a state of unresponsiveness or exhaustion which could be reversed by blocking the ligand PD-L1 (28). Therefore, we injected blocking anti-PD-L1 antibodies before and during LCMV infection to determine whether CD8 T_ML_ cell function could be restored. However, when mice were analyzed on day 10 after infection, we observed that the frequency and total number of polyfunctional IFN-γ and TNF-α producing CD8 T cells did not increase in comparison to isotype-treated animals (Figures 5A,B). This indicates that either other inhibitory checkpoints are involved in dampening the responsiveness of CD8 T_ML_ cells in CD4Cre/R-DTA mice or that the functionality of these cells cannot be restored by extrinsic signals. For reasons that remain unclear at this point we further observed increased viral loads in spleen, liver, and kidney of anti-PD-L1-treated as compared to isotype control-treated CD4Cre/R-DTA mice (Figure 5C).

### Transfer of Naïve Polyclonal CD4 T Cells Is Sufficient to Restore CD8 T_ML_ Functionality in CD4Cre/R-DTA Mice

**Figure 6:**
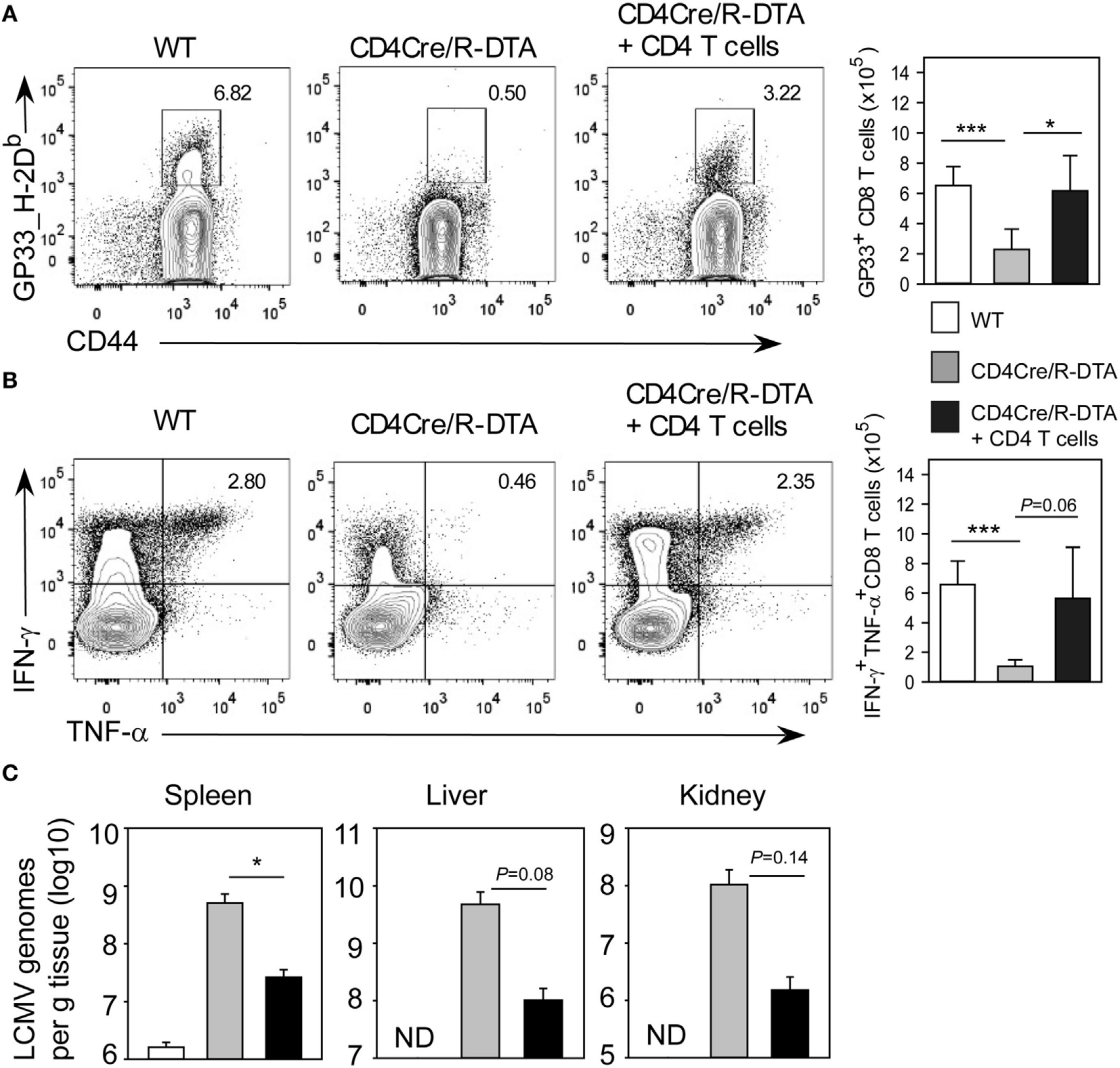
**Transfer of naïve CD4 T cells is sufficient to restore CD8 memory-like T cells functionality in CD4Cre/R-diphtheria toxin alpha (DTA) mice**. CD4Cre and CD4Cre/R-DTA mice were infected with LCMV. One group of CD4Cre/R-DTA mice received 3 × 10^6^ polyclonal naïve CD4 T cells from normal CD45.1^+^CD45.2^+^ heterozygous congenic mice 1 day before infection. Analysis took place on day 14 after infection. **(A)** Contour plots show stainings with gp33_H2-D^b^ dextramer on gated CD8 T cells. Bar graph depicts the mean number + SEM of gp33-specific CD8 T cells in lymphocytic choriomeningitis virus (LCMV)-infected CD4Cre (white), CD4Cre/R-DTA mice (gray), and CD4Cre/R-DTA mice with CD4 T cell transfer (black). **(B)** Intracellular staining of IFN-γ and TNF-α in gated CD8 T cells restimulated with gp33 peptide. Bar graph depicts the mean number + SEM of IFN-γ and TNF-α-producing CD8 T cells in LCMV-infected CD4Cre (white), CD4Cre/R-DTA mice (gray), and CD4Cre/R-DTA mice with CD4 T cell transfer (black). **(C)** Copy numbers of viral genomes in spleen, liver, and kidney of LCMV-infected CD4Cre (white), CD4Cre/R-DTA mice (gray), and CD4Cre/R-DTA mice with CD4 T cell transfer (black) were determined by quantitative RT-PCR. ND, not detectable. A total of 8–14 mice per group from 5 experiments. *P < 0.05; ***P < 0.001 by Mann–Whitney *U*-test **(A,B)** or Student’s *t*-test **(C)**.

To further investigate whether functionality in CD8 T_ML_ cells can be restored *in vivo*, we transferred 3 × 10^6^ purified polyclonal CD4 T cells from congenic CD45.1^+^CD45.2^+^ heterozygous mice into CD4Cre/R-DTA mice with the idea to provide “helper” cells that could mediate activation of endogenous CD8 T_ML_ cells. These reconstituted mice were infected with LCMV and analyzed on day 14 after infection. The transferred CD45.1^+^CD45.2^+^ CD4 T cells constituted about 60% of all CD4 T cells and 6% of them coexpressed IFN-γ and TNF-α after gp61 restimulation (Figure S2 in Supplementary Material). By contrast, only 0.8% of the endogenous CD4 T_ML_ cells produced both cytokines indicating a functional impairment of these cells. With regard to the CD8 T cell response, CD4Cre/R-DTA mice that had received CD4 T cells showed an increased frequency and total number of gp33_H-2D^b^ dextramer positive and IFN-γ/TNF-α polyfunctional CD8 T cells as compared to non-reconstituted mice (Figures 6A,B). In addition, antiviral activity was partially restored (Figure 6C). This indicates that CD8 T_ML_ cells require help by CD4 T cells and endogenous CD4 T_ML_ cells in CD4Cre/R-DTA mice are not sufficient for this helper function. However, transferred CD4 cells from naïve wild-type mice could efficiently restore the functional capacity of CD8 T_ML_ cells in CD4Cre/R-DTA mice and thereby promote the antiviral immune responses.

## Discussion

The participation of lymphopenia-induced T_ML_ in protective immune responses remains unclear although the mechanisms of T_ML_ development are quite well understood (1). Functional characterization of T_ML_ cells *in vivo* is critical to elucidate mechanisms that could improve vaccinations of people where T_ML_ cells represent a dominant population such as the elderly population, AIDS patients, or people recovering from bone marrow transplantations.

Using constitutively T cell-lymphopenic CD4Cre/R-DTA mice we demonstrate here that CTLs developed from CD8 T_ML_ cells and efficiently killed transferred target cells but could not control viral replication during low-dose acute LCMV infection. In addition, expansion of T_FH_ cells, germinal center formation, and antibody production was significantly impaired in CD4Cre/R-DTA mice. However, the impaired CTL functionality could be reversed by transfer of naïve polyclonal CD4 T cells from wild-type mice highlighting the functional plasticity of these cells which might be exploited for future vaccination strategies or therapeutic options in chronic viral infections.

It is important to note that the T cells which escape deletion in CD4Cre/R-DTA mice show a normal survival, turnover, and proliferation *in vitro* and *in vivo* excluding the possibility that they are short-lived or generally unresponsive cells (22). Yet, we observed a poor T cell response in CD4Cre/R-DTA mice during acute LCMV induction. By analysis of the Vβ chain usage we previously showed that the T cell receptor repertoire diversity is reduced in CD4Cre/R-DTA mice which indicates that only few thymic emigrants escaped the deletion and these cells retained the loxP-flanked STOP cassette in front of the DTA gene (22). The number of LCMV gp33_H-2D^b^-specific CD8 T cells in a naïve C57BL/6 mouse has been calculated to range between 300 and 1,000 (29, 30). Others provided evidence that more than 1,000 clonotypic TCRs for this particular peptide-MHC complex exist in the naïve repertoire of C57BL/6 mice (31). Assuming that lymphopenic conditions do neither inhibit nor favor expansion of gp33_H-2D^b^-specific CD8 T cells *per se* one can estimate that only about 100 of these cells are present in naïve CD4Cre/R-DTA mice and most of them probably acquired a T_ML_ phenotype due to homeostatic proliferation. Despite their low number and altered phenotype, they massively expanded to constitute about 2.5 × 10^5^ cells in the spleen on day 8 after infection and they efficiently killed transferred target cells within 90 min *in vivo*. However, the viral titers remained high and the infection could not be controlled. This finding suggests that CTLs in CD4Cre/R-DTA mice were functionally impaired. On a single-cell level we observed fewer CTLs that expressed IFN-γ together with TNF-α after *in vitro* restimulation. It has been demonstrated that so-called polyfunctional T cells which express IFN-γ together with IL-2 and/or TNF-α are much more potent effector cells in mice and humans as compared to single cytokine producers (32, 33). However, mice that lack receptors for IFN-γ or TNF-α can still control acute LCMV infection indicating that other factors derived from polyfunctional CD8 T cells might be required to reduce viral titers in organs (34, 35).

The lower frequency of polyfunctional T cells could be linked to the expression of the senescence marker KLRG1 and the inhibitory receptor PD-1 on a large proportion of T cells in naïve CD4Cre/R-DTA mice. We have previously shown that KLRG1 is expressed on mouse and human T cells after activation when they reach replicative senescence (36, 37). It is well known that PD-1 is expressed on effector T cells but also on exhausted T cells (28). However, functional exhaustion of CTLs is also observed with cells from PD-1-deficient mice (38). PD-1 was further found to be expressed on the surface of dysfunctional homeostatically expanded T cells after adoptive transfer into lymphopenic hosts (27). Interestingly, the function of PD-1^+^ T cells could be restored by anti-PD-1 or anti-PD-L1 checkpoint inhibitors as demonstrated in a chronic LCMV clone 13 infection model (28). Further, blockade of another inhibitory receptor, Tim-3, or injection of IL-2 in combination with anti-PD-L1 showed improved reactivation of LCMV-specific CD8 T cells in the chronic LCMV clone 13 model (39, 40). However, as we show here PD-L1 blockade during acute LCMV-WE infection which becomes chronic in CD4Cre/R-DTA mice did not restore expansion of polyfunctional T cells and the infection could not be controlled.

Importantly, expansion, cytokine production, and antiviral activity of LCMV-specific CD8 T cells could be improved by transfer of 3 × 10^6^ polyclonal CD4 T cells into CD4Cre/R-DTA mice. This indicates that LCMV-specific CD8 T cells are not *per se* missing in CD4Cre/R-DTA mice but they are functionally impaired and a relatively low number of transferred naïve CD4 T cells but not endogenous T_ML_ CD4 T cells were sufficient to revive these CD8 T cells. Future studies will have to identify the critical reviving factors provided by the transferred CD4 T cells to elucidate the mechanisms that regulate functional plasticity of T_ML_ cells *in vivo*. A better understanding of T_ML_ plasticity and effector functions will help to develop more efficient vaccines for the elderly population and perhaps boost antiviral CD8 T cell responses under lymphopenic conditions including AIDS patients or CMV patients after bone marrow transplantation.

## Author Contributions

MS, SS, and DV designed experiments. MS and SS performed experiments. MS, SS, and DV wrote the manuscript.

## Conflict of Interest Statement

The authors declare that the research was conducted in the absence of any commercial or financial relationships that could be construed as a potential conflict of interest.
