# Supplementary material for: CD4 T Helper Cells Instruct Lymphopenia-Induced Memory-Like CD8 T Cells for Control of Acute LCMV Infection
Source: Front Immunol. 2016 Dec 21;7:622. doi: 10.3389/fimmu.2016.00622 (PMC5174106; doi:10.3389/fimmu.2016.00622)
Supplement: Supplementary file 1 [file Image_1.PDF]

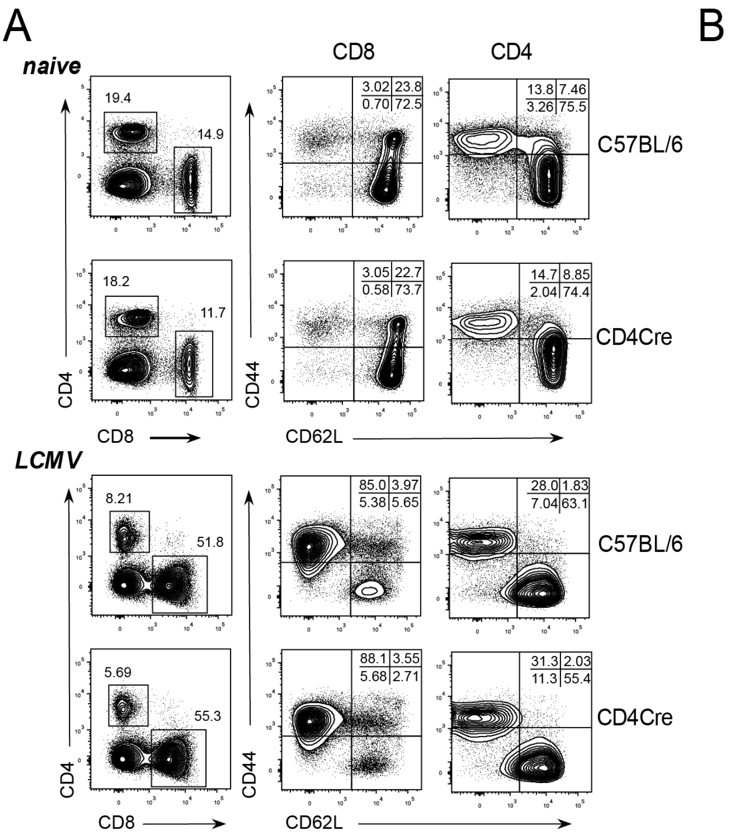

Supplemental Figure 1. CD4Cre mice show a normal T cell response to LCMV infection. A) Contour plots show the frequency of CD4 and CD8 T cells (left) and their activation status (right) in the spleens of naïve (top) and day 8 LCMV-infected (bottom) C57BL/6 and CD4Cre mice. B) Frequency of gp33-specific CD8 T cells and gp66-specific CD4 T cells in the spleens of day 8 LCMV infected C57BL/6 and CD4Cre mice. Plots are representative for 3 mice per mouse strain.

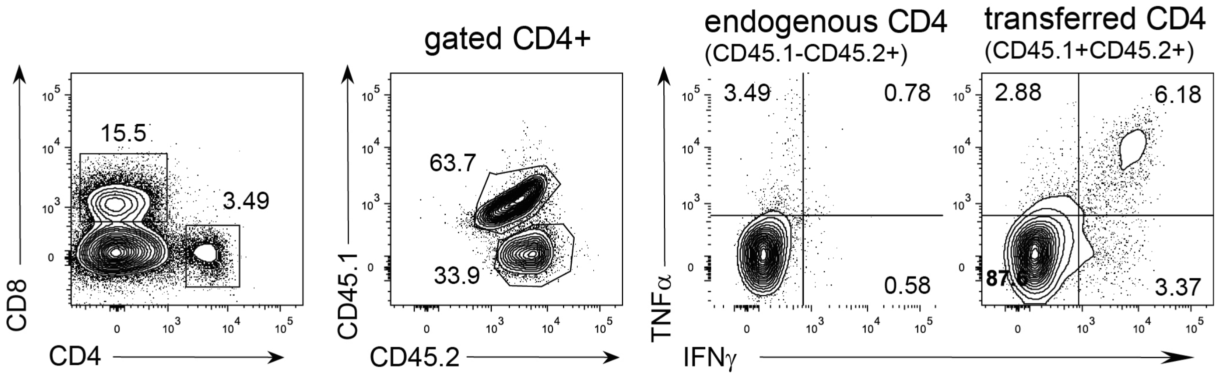

Supplemental Figure 2. Cytokine expression in endogenous and transferred CD4 T cells. CD4Cre/R-DTA mice were reconstituted with $3x10^6$ naïve CD4 T cells from CD45.1 congenic mice one day before LCMV infection. Splenocytes were re-stimulated with gp61 peptide on day 14 after infection and analyzed for intracellular cytokine production. The contour plot of gated CD4 T cells shows the frequency of endogenous ($CD45.2^+CD45.1^-$) and transferred ($CD45.2^+CD45.1^+$) CD4 T cells. The plots on the right show the frequency of TNF-α- and IFN-γ-producing CD4 T cells within the endogenous and transferred CD4 T cells.
